# Priorisierung intensivmedizinischer Behandlungsplätze – Konzeptvorschlag

**DOI:** 10.1007/s00104-020-01334-0

**Published:** 2021-01-06

**Authors:** A. C. Deppe, F. Kolibay, V. Burst, S. Simon, M. Rothschild, M. Kochanek, T. Annecke, C. Adler, F. Dusse, M. Hof, G. Langebartels, S. Reimers, S. Muckel, B. Roth, J. Wolff, O. A. Onur

**Affiliations:** 1 Herzchirurgische Intensivstation, Klinik und Poliklinik für Herzchirurgie, herzchirurgische Intensivmedizin und Thoraxchirurgie, Universität zu Köln, Medizinische Fakultät und Uniklinik, Köln, Deutschland; 2 Katastrophenschutzbeauftragter, Stabsabteilung Klinikangelegenheiten und Krisenmanagement des Ärztlichen Direktors, Universität zu Köln, Medizinische Fakultät und Uniklinik, Köln, Deutschland; 3 Zentrale Notaufnahme, Klinik II für Innere Medizin: Nephrologie, Rheumatologie, Diabetologie und Allgemeine Innere Medizin, Universität zu Köln, Medizinische Fakultät und Uniklinik, Köln, Deutschland; 4 Zentrum für Palliativmedizin, Universität zu Köln, Medizinische Fakultät und Uniklinik, Köln, Deutschland; 5 Institut für Rechtmedizin, Universität zu Köln, Medizinische Fakultät und Uniklinik, Köln, Deutschland; 6 Internistische Intensivstation, Klinik I für Innere Medizin: Hämatologie und Onkologie, Universität zu Köln, Medizinische Fakultät und Uniklinik, Köln, Deutschland; 7 Anästhesiologische Intensivstation, Klinik für Anästhesiologie und operative Intensivmedizin, Universität zu Köln, Medizinische Fakultät und Klinikum der Universität zu Witten/Herdecke, Kliniken Köln, Köln, Deutschland; 8 Kardiologische Intensivstation, Klinik III für Innere Medizin: Allgemeine und interventionelle Kardiologie, Elektrophysiologie, Angiologie, Pneumologie und internistische Intensivmedizin, Universität zu Köln, Medizinische Fakultät und Uniklinik, Köln, Deutschland; 9 Neurochirurgische Intensivstation, Klinik und Poliklinik für allgemeine Neurochirurgie, Universität zu Köln, Medizinische Fakultät und Uniklinik, Köln, Deutschland; 10 Ärztlicher Koordinator Intensivmedizin, Stabsabteilung Klinikangelegenheiten und Krisenmanagement des Ärztlichen Direktors, Universität zu Köln, Medizinische Fakultät und Uniklinik, Köln, Deutschland; 11 Pflegedienstleitung Intensivpflege, Universität zu Köln, Medizinische Fakultät und Uniklinik, Köln, Deutschland; 12 Juristische Fakultät, Öffentliches Recht und Religionsrecht, Institute für Religionsrecht, Universität zu Köln, Köln, Deutschland; 13 Klinik und Poliklinik für Kinder- und Jugendmedizin, Universität zu Köln, Medizinische Fakultät und Uniklinik, Köln, Deutschland; 14 Katholische Seelsorge, Universität zu Köln, Medizinische Fakultät und Uniklinik, Köln, Deutschland; 15 Neurologische Intensivstation, Klinik und Poliklinik für Neurologie, Universität zu Köln, Medizinische Fakultät und Uniklinik, Köln, Deutschland

**Keywords:** Stufenmodell, Priorisierung, Triage-Team, Notfallversorgung, Prognose, Stage model, Prioritization, Triage team, Emergency care, Prognosis

## Abstract

In der Situation des Beatmungsbettenmangels sind ethisch begründbare, transparente und nachvollziehbare Entscheidungen zu treffen. Dieses Konzept sieht vor, dass zunächst alle Patienten nach Notwendigkeit intubiert und dann von einem Triage-Team beurteilt werden. Dabei stehen neu aufgenommene COVID-Patienten mit neu aufgenommenen Nicht-COVID-Patienten und bereits intensivmedizinisch behandelte Patienten in Konkurrenz um ein Beatmungsgerät. Die Kombination der kurz- und langfristigen Prognose soll dem interprofessionellen Triage-Team ermöglichen, nachvollziehbare Entscheidungen zu treffen. Ziel des Priorisierungskonzeptes ist es, möglichst viele Menschenleben zu retten und die Behandlungsteams von der schwierigen Entscheidung der Priorisierung zu entlasten.

## Abstract (en)

In the situation of a shortage of ventilation beds, ethically justifiable, transparent and comprehensible decisions must be made. This concept proposes that all patients are first intubated depending on necessity and then assessed by a triage team afterwards. In this situation newly admitted COVID patients compete with newly admitted Non-COVID patients as well as patients already treated in intensive care units for a ventilator. The combination of short-term and long-term prognoses should enable the interprofessional triage team to make comprehensible decisions. The aim of the prioritization concept is to save as many human lives as possible and to relieve the treatment team of the difficult decision on prioritization.

Dieses Konzept zur Priorisierung intensivpflichtiger Patienten aufgrund eines pandemiebedingten Ressourcenmangels ist in sehr konstruktiver Zusammenarbeit mit Vertretern der Pflegeleitung, der Notaufnahme, der Intensivstationen verschiedener Disziplinen, der Palliativmedizin, der Seelsorge und dem Klinikvorstand entstanden. Das Ethikkonsil hat nach Prüfung und Diskussion der ethischen und rechtlichen Aspekte für das Konzept ein positives Votum abgegeben. Über die Erstellung des Konzeptes hinaus, eint uns die Hoffnung, dass dieses Priorisierungskonzept nie zur Anwendung kommen mag.

Im Rahmen der SARS-CoV-2(„severe acute respiratory syndrome coronavirus typ 2“)-Pandemie waren 2020 in zahlreichen Städten und Regionen weltweit sogar trotz kurzfristig erweiterter Behandlungskapazitäten die Ressourcen, insbesondere an Beatmungsplätzen, zur Behandlung des Patientenaufkommens nicht ausreichend. Im Massenanfall beatmungsbedürftiger Patienten sahen sich Mediziner mit der Entscheidung konfrontiert, welche Patienten zu behandeln sind bzw. bei welchen auf eine Behandlung zu verzichten ist. Zahlreiche Ärzte aus Notaufnahmen und Intensivstationen berichten von Entscheidungen basierend auf dem Alter der Patienten und/oder bestehender Vorerkrankungen und einer damit angenommenen schlechten Prognose. In vielen Fällen scheint auch der Zufall, wer eine der letzten Behandlungsmöglichkeiten in Anspruch nehmen kann, zu entscheiden. Es ist anzunehmen, dass in vielen Fällen die ärztlichen Entscheidungen in Phasen mit sehr hoher Arbeitsbelastung von Einzelnen getroffen, vertreten und durchgeführt wurden. Diese überfordernde Verantwortung wird nicht selten zu Belastungen führen, die schwerlich auszuhalten sind. Aus diesen Erfahrungen lernend, wurden Empfehlungen der Deutschen interdisziplinären Vereinigung für Intensiv- und Notfallmedizin (DIVI) im Zusammenschluss verschiedener Fachgesellschaften veröffentlicht [3]. In der Literatur findet man nur sehr wenig und v. a. noch nie angewendete Konzepte zur Priorisierung [1, 3, 5, 6].

Die Kriterien zeigen erfreulicherweise über nationale und fachliche Grenzen hinweg einen gewissen „common sense“. Um die Empfehlungen für die Praxis in einer pandemischen Ausnahmesituation anwendbar zu gestalten, wird dieses hier vorgestellte Konzept für die Anwendung konkretisiert.

## Grundsätze

Grundsätzlich wird trotz einer zu erwartenden Ressourcenknappheit das Ziel verfolgt, möglichst vielen Patienten das Überleben zu gewährleisten. Dieses Ziel soll dadurch erreicht werden, dass Patienten mit einer guten klinischen Erfolgsaussicht priorisiert werden. Hierzu werden die kurzfristige Prognose und die langfristige Prognose eingeschätzt und in einem Score kombiniert. So werden Patienten priorisiert, die in einem verhältnismäßig besseren klinischen Zustand das Krankenhaus erreichen und damit bei adäquater Behandlung eine bessere Überlebensprognose im Vergleich zu Patienten in einem sehr schlechten Zustand haben. Auf der anderen Seite werden Patienten priorisiert, die keine oder wenig Vorerkrankungen und konsekutiv eine bessere Prognose haben.

Das Lebensalter des Patienten spielt in dieser Betrachtungsweise per se keine Rolle. Jedwede Berücksichtigung des Alters als Kriterium ohne Betrachtung der individuellen Situation ist aus Sicht der Autoren ethisch nicht vertretbar, rechtlich nicht haltbar und widerspricht dem medizinischen Handeln außerhalb von Pandemiezeiten. Durch die Berücksichtigung der Vorerkrankungen sind ältere Patienten im Mittel innerhalb dieses Konzeptes naturgegeben schlechter gestellt, einen zusätzlichen Malus durch die Hinzunahme des Alters oder eines Scores zur Gebrechlichkeit (z. B. Frailty Scale) stellt aus Sicht der Autoren eine übermäßige Benachteiligung und damit eine Diskriminierung dar. Altersgrenzen sind zudem arbiträr. Neben dem hohen Lebensalter soll der soziale Status, der gesellschaftliche Nutzen, der finanzielle Beitrag und die Zugehörigkeit zu einer bestimmten Gruppe ebenfalls weder in der einen noch in der anderen Richtung eine Rolle spielen [2]. Ausnahmen von diesem Konzept gelten zum einen für Kinder, die von der Priorisierung gänzlich ausgenommen werden. Zum anderen werden Schwangere mit intakter Schwangerschaft bevorzugt behandelt, auch wenn der Fetus in diesem Kontext nicht der zu behandelnde Patient ist.

Ein diskutiertes Szenario, in dem in vorhereilendem Gehorsam ein Patient mit schlechter Gesamtprognose bei noch verfügbarer Kapazität gar nicht behandelt wird, um diesen Behandlungsplatz prophylaktisch für einen potenziellen zukünftigen Patienten frei zu halten, widerspricht aus der Sicht der Autoren medizinischen und ethischen Grundsätzen.

Es gilt zu betonen, dass die im Weiteren beschriebenen Kriterien nicht rein auf COVID(„corona virus disease“)-Patienten Anwendung finden dürfen, sondern das gesamte Patientenkollektiv des Krankenhauses umfasst. Anders ausgedrückt: COVID-Patienten und Nicht-COVID-Patienten werden gleich behandelt. Aus diesem Grund sind auch Operationen, die nach dem Eingriff eine intensivmedizinische Betreuung nach sich ziehen, explizit in die Triagierung einzubeziehen. Priorisiert werden in diesem Zusammenhang einerseits Eingriffe, die bei Unterlassen kurzfristig zu einer schlechten Prognose bei dem betroffenen Patienten führen würden. Andererseits werden Operationen priorisiert, die nur eine kurze Nachbetreuung auf der Intensivstation nach sich ziehen.

Zu guter Letzt war es den Autoren ein Anliegen, dass der Zufall gar keine Rolle spielen soll. Inakzeptabel sind Lotterien, die auch nicht durch den Zeitpunkt des Eintreffens des Patienten (im Sinne von „first come, first served“) verursacht sein dürfen.

## Voraussetzungen

Aus Sicht der Autoren sind drei wichtige Aspekte vorab zu bedenken und zu klären:

### Erster Aspekt.

Das hier vorgestellte Konzept sollte vor der Katastrophensituation bekannt gemacht werden und wenn nötig vorab den lokalen und regionalen Umständen angepasst und modifiziert werden. Auch wenn es einem widerstrebt, sich mit solch einem Szenario auseinanderzusetzen und in den hoffentlich allermeisten Fällen die befürchtete Situation nicht eintritt, ist es essenziell, für alle Eventualitäten vorbereitet zu sein, um Situationen der Überforderungen und Verzweiflung zu verhindern.

### Zweiter Aspekt.

Das Priorisierungskonzept soll Anwendung in einer Situation der Ressourcenknappheit finden. Die Knappheit von z. B. Beatmungsplätzen sollte von übergeordneter Stelle (Ärztlicher Direktor, Kommunal‑, Landes- oder Bundesbehörde) und unabhängig von ökonomischen Entscheidungen festgestellt worden sein. Gleichzeitig wird gefordert, dass auch in der Phase mangelnder Ressourcen regional und überregional Verlegungsoptionen aktiv evaluiert werden. Es wäre nicht zu vertreten, dass in einer Klinik Patienten aufgrund unterlassener Behandlung zu Schaden kommen, wohingegen in Kliniken, die zeitgerecht und mit einer medizinischen Versorgungsoption zu erreichen wären, Kapazitäten bestehen. Hier wäre sicher hilfreich, wenn im Katastrophenfall die fallpauschalierte Vergütung unkompliziert durch eine für den Zeitraum garantierte, kostendeckende Vergütung ersetzt würde. Somit wären ökonomische Überlegungen im Priorisierungskonzept unwirksam.

### Dritter Aspekt.

Als letzte Voraussetzung sollte gewährleistet sein, dass umliegende Kliniken nach vergleichbaren Grundsätzen handeln. Es wäre z. B. nicht sinnvoll, wenn in einer Klinik Patienten mit einer Lebenserwartung unter 12 Monaten grundsätzlich nicht behandelt werden und in einer Nachbarklinik schon. Um solche Unterschiede zu unterbinden, wäre eine Abstimmung auf regionaler und sogar überregionaler Ebene zwischen Krankenhäusern und Rettungsdiensten wünschenswert, notwendig, wenn nicht sogar unabdingbar.

## Entscheidungsalgorithmus im Stufenmodell

**Figure 1:**
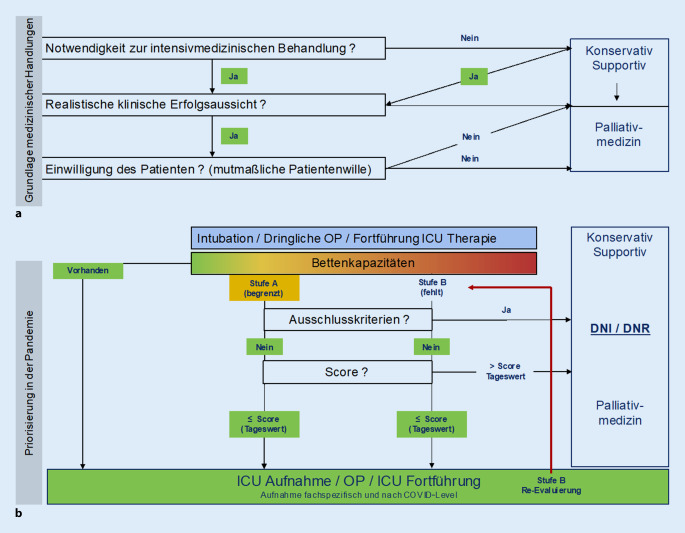

In der Pandemie müssen Pläne und Protokolle bei knappen Ressourcen von einer individuellen Perspektive der Patientenversorgung zu einem bevölkerungsbezogenen Fokus übergehen. Die Zuweisung von Ressourcen muss dabei prinzipiell erfolgen und versuchen, Gerechtigkeit und Fairness zu gewährleisten [3, 7]. Dennoch sollte dem einzelnen behandelnden Arzt zu jeder Zeit und in jedem denkbaren Szenario die Möglichkeit gegeben sein, seinen Patienten zunächst nach allen Regeln medizinischen Handelns („good clinical practice“) betreuen zu können. Dazu zählt zunächst, zu eruieren, ob überhaupt die Indikation, eine realistische Erfolgsaussicht und der Wille des Patienten für eine intensivmedizinische Behandlung gegeben sind (Abb. 1). Sind die Voraussetzungen erfüllt oder können aufgrund der dringlichen Behandlungsnotwendigkeit kurzfristig nicht erhoben werden, wird eine Therapie indikationsgerecht begonnen. Die Evaluation der Behandlungskapazitäten erfolgt nach Abschluss der notfallversorgenden Erstmaßnahmen.

Stehen Betten zur Verfügung, wird der Patient auf die Intensivstation verlegt. Ein prophylaktisches Freihalten und damit Vorenthalten des Platzes für einen noch nicht bekannten Patienten läuft dem Ziel, möglichst viele Patienten zu retten, zuwider.

Das Priorisierungskonzept findet Anwendung für alle Patienten, die eine knappe Ressource benötigen. Diese Knappheit von z. B. Beatmungsplätzen sollte so weit möglich von übergeordneter Stelle (Ärztlicher Direktor, Kommunal‑, Landes- oder Bundesbehörde) festgestellt worden sein. Gleichzeitig wird gefordert, dass auch in der Phase mangelnder Ressourcen regional und überregional Verlegungsoptionen aktiv evaluiert werden. Zur Durchführung der Priorisierung wird die Bildung eines interprofessionellen Triage-Teams ohne hierarchische Abhängigkeiten bestehend aus zwei Oberärzten unterschiedlicher Abteilungen (mit der Zusatzbezeichnung Intensivmedizin oder Notfallmedizin), einem Beauftragten des Ärztlichen Direktors, einem Vertreter des Ethikkonsils und einem Vertreter des Pflegedienstes (Gesundheits- und Fachkrankenpfleger für Intensivpflege und Anästhesie) gefordert. Entscheidungen sollen möglichst im Konsens getroffen werden. Bei Dissens sollte der Klinikdirektor der betroffenen Abteilung als Mediziner mit der größten Expertise und als Ranghöchster in die Entscheidung einbezogen werden und letztlich eine Entscheidung treffen.

Unterschieden werden zwei Stufen der Priorisierung:

In Stufe A (Tab. 1) kommt es zu einem Massenanfall an Schwerkranken bzw. Schwerverletzten oder es herrscht bereits eine Ressourcenknappheit, sodass Patienten bereits in die extra für die Pandemie zusätzlich geschaffenen Intensivkapazitäten aufgenommen werden. Sollte die Zahl der zu behandelnden Patienten die Zahl der Behandlungsplätze überschreiten, werden Patienten mit schwersten Begleiterkrankungen und damit assoziierter schlechter Behandlungsaussicht einer intensivmedizinischen Behandlung nicht mehr zugeführt. Die Ausschlusskriterien (Tab. 2) für eine intensivmedizinische Behandlung können aufgrund von Erfahrungs- und Erkenntnisgewinn während der Pandemie bzw. Anwendung des Priorisierungskonzeptes angepasst werden.

**Table 1:** 

Stufe A	Betten für die Intensivpflege verfügbar, aber begrenzte Kapazitäten
	⇨ Aufnahme- und Interventions-Triage
Schritt A1)	Überprüfung der Ausschlusskriterien (s. Tab. 2)
	*⇨ wenn zutreffend, keine ICU-Therapie*
Schritt A2)	Punktesystem
	*⇨ wenn Wert über Cut-off oder im Vergleich zweier Patienten der Wert höher, dann keine ICU-Therapie*

*IUC* „intensive care unit“

**Table 2:** 

Fehlender (mutmaßlicher) Patientenwille für Intensivmedizin
Unbeobachteter Kreislaufstillstand, wiederkehrender Kreislaufstillstand, Kreislaufstillstand ohne ROSC
Fortgeschrittene, metastasierende Krebserkrankung ohne Therapieoptionen
Neurodegenerative Erkrankung im Endstadium
Schwere und irreversible zentralneurologische Beeinträchtigung
*Chronische Krankheit:*
– Stadium NYHA IV Herzinsuffizienz
– COPD Stufe C oder D und zusätzlich FEV1 <25 % oder PHT oder Sauerstoffbehandlung
– Zirrhose nach Child-Pugh >8
Schwere Kreislaufinsuffizienz, die trotz Erhöhung der Vasoaktiva therapieresistent ist (Hypotonie und/oder anhaltende Organminderperfusion)
Schwere Verbrennungen: >65 Jahre oder >50 % der KOF betroffen
Erwartete Überlebensdauer <12 Monate

Aufgelistet sind die Ausschlusskriterien, die in Stufe A bei allen neu aufzunehmenden Patienten und in Stufe B bei allen neu aufzunehmenden und bei allen Patienten mit einer intensivmedizinischen Therapiedauer über 72 h zu evaluieren sind. Patienten mit einem Ausschlusskriterium werden, wenn möglich konservativ behandelt, ansonsten palliativmedizinisch weiterbetreut

*COPD* „chronic obstructive pulmonary disease“, *FEV1* „forced expiratory pressure in 1 s“, *KOF* Körperoberfläche, *NYHA* New York Heart Association, *PHT* pulmonaler Hypertonus, *ROSC* „return of spontaneous circulation“

In Stufe B (Tab. 3) sind alle Beatmungsbetten auf nicht absehbare Zeit belegt. Zu diesem Zeitpunkt konkurrieren neu aufgenommene Patienten, Patienten mit einer dringlichen Operationsindikation und beatmete Patienten auf der Intensivstation um ein Beatmungsgerät. Die Ausschlusskriterien sind in dieser Situation auch bei beatmeten Patienten auf der Intensivstation zu überprüfen. Konkurrieren nach Anwendung der Ausschlusskriterien noch mehr Patienten als Kapazitäten vorhanden sind, wird zur Priorisierung die kurz- und langfristige Prognose auf einen möglichen Behandlungserfolg ausschlaggebend. Die kurzfristige Prognose konservativer Patienten für das Überleben wird anhand des SOFA(Sequential Organ Failure Assessment Score)-Scores [8] und die langfristige Prognose für das Überleben anhand der Komorbiditäten in einem Punktesystem ermittelt (Tab. 4). Für chirurgische Patienten mit postoperativer intensivmedizinischer Behandlung wird äquivalent die Dringlichkeit der Operation für die kurzfristige Prognose erfasst. Hier ist zu fordern, dass äquivalent zum Leitfaden für maligne thorakale Erkrankungen [4] für die vorhandenen chirurgischen Fächer das Punktesystem mit konkreten Definitionen gefüllt wird. Ein Eingriff, der einen kurzen Intensivstationsaufenthalt (≤48 h) erwarten lässt, wird durch Punktabzug (Tab. 5) priorisiert. Frauen mit intakter Schwangerschaft werden ebenfalls durch Punktabzug sowohl beim Scoring der konservativen als auch der chirurgischen Patienten bevorzugt, da man mit der Behandlung der Patientin potenziell zwei Leben retten könnte.

**Table 3:** 

Stufe B	Keine verfügbaren Intensivpflegebetten
	Keine inhäusige Reanimation mehr
	Nur noch Operationen bei einem Punkt, tägliche Reevaluation
	Ressourcenmanagement durch Entscheidungen über Behandlungsabbruch bei ICU-Patienten:
B1)	Überprüfung der „Ausschlusskriterien“ (s. Tab. 2)
	*⇨ wenn zutreffend, Abbruch ICU-Therapie*
B2)	Punktesystem (s. Tab. 4 und 5)
	*⇨ wenn Wert über Cut-off oder im Vergleich zweier Patienten der Wert höher, dann keine ICU-Therapie*
B3)	Überprüfung Zustandsänderung der letzten 24 h an allen ICU-Patienten frühestens 72 h nach ICU-Aufnahme
	a. Auftreten eines Herzstillstands während des Aufenthalts
	b. Zusätzlich zur Lunge Versagen zweier weiterer Organe
	c. Signifikante Erhöhung des SOFA-Scores (>2 Punkte)
	d. Keine Verbesserung der respiratorischen oder hämodynamischen Bedingungen *⇨ wenn zutreffend, Abbruch ICU-Therapie*

*IUC* „intensive care unit“, *SOFA* Sequential Organ Failure Assessment Score

**Table 4:** 

Prinzip	Spezifikation	Punktesystem^a^			
		1	2	3	4
*Prognose für kurzfristiges Überleben*	*SOFA*	SOFA ≤8	SOFA ≤9–11	SOFA ≤12–11	SOFA >14
*Prognose für langfristiges Überleben*	*Komorbiditäten*	–	–	Schwere Komorbidität	–
**Schwere Komorbiditäten**					
Ausgedehnte Verbrennungen (>40 % BSA) mit Inhalation					
Schwere zerebrale Defizite nach Stroke (mRS 5)					
Mittelschwere Demenz (CD ≥2)					
Schweres Polytrauma mit persistierendem Schockzustand trotz Intervention					
Chronische Krankheit:					
– Stadium NYHA III oder IV Herzinsuffizienz					
– COPD-C- oder -D-Stadium oder FEV1 <25 % oder PHT oder Sauerstoffbehandlung zu Hause					
– Zirrhose nach behandlungsrefraktärer Aszites oder Enzephalopathie > Stadium I					
– Chronisches Nierenversagen Stadium V					
Geschätzte Überlebensdauer <24 Monate					

Dargestellt ist der Score, der bei allen Patienten ohne Operationsindikation mit Notwendigkeit einer intensivmedizinischen Behandlung zur Anwendung kommt. Die kurzfristige Prognose wird über den SOFA-Score ermittelt und in einen Score-Punkt umgewandelt. Für schwere Komorbiditäten werden 3 Punkte vergeben. Je niedriger der Score umso eher Priorisierung zur Aufnahme auf die Intensivstation

*BSA* „“body surface area, *CD* „“clinical dementia, *COPD* „chronic obstructive pulmonary disease“, *FEV1* „forced expiratory pressure in 1 s“, *mRS* modifizierte Rankin-Skala, *NYHA* New York Heart Association, *PHT* pulmonaler Hypertonus, *SOFA* Sequential Organ Failure Assessment Score

^a^Punktabzug für Schwangere mit intakter Schwangerschaft

**Table 5:** 

Punktesystem für Operation (je weniger Punkte, desto eher Operation)					
Prinzip	Spezifikation	Punktesystem^a, b^			
		1	2	3	4
*Prognose für kurzfristiges Überleben*	*Operationsdringlichkeit*	Notfall, Mortalität oder sehr schlechtes Outcome innerhalb einer Woche	Schlechtes Outcome bei Verzögerung >4 Wochen	Schlechte Outcome bei Verzögerung 4–12 Wochen	Schlechtes Outcome bei Verzögerung >12 Wochen
*Prognose für langfristiges Überleben*	*Komorbiditäten*	–	–	Schwere Komorbidität	–
**Schwere Komorbiditäten**					
Ausgedehnte Verbrennungen (>40 % BSA) mit Inhalation					
Schwere zerebrale Defizite nach Stroke (mRS 5)					
Mittelschwere Demenz (CD ≥2)					
Schweres Polytrauma mit persistierendem Schockzustand trotz Intervention					
Chronische Krankheit:					
– Stadium NYHA III oder IV Herzinsuffizienz					
– COPD-C- oder -D-Stadium oder FEV1 <25 % oder PHT oder Sauerstoffbehandlung zu Hause					
– Zirrhose nach behandlungsrefraktärer Aszites oder Enzephalopathie > Stadium I					
– Chronisches Nierenversagen Stadium V					
Geschätzte Überlebensdauer <24 Monate					

Dargestellt ist der Score, der bei allen Patienten mit Operationsindikation und postoperativer Notwendigkeit einer intensivmedizinischen Behandlung zur Anwendung kommt. Bei gegebener Operationsindikation wird das Risiko einer Zustandsverschlechterung aufgrund der Operationsverzögerung erhoben und in einen Punkt für die kurzfristige Prognose umgewandelt. Für schwere Komorbiditäten werden 3 Punkte vergeben. Je niedriger der Score umso eher Priorisierung zur Aufnahme auf die Intensivstation

*BSA* „“body surface area, *CD* „“clinical dementia, *COPD* „chronic obstructive pulmonary disease“, *FEV1* „forced expiratory pressure in 1 s“, *mRS* modifizierte Rankin-Skala, *NYHA* New York Heart Association, *PHT* pulmonaler Hypertonus

^a^Punktabzug für Schwangere mit intakter Schwangerschaft

^b^Punktabzug für Eingriffe, die eine kurze Nachbetreuung auf Intensivstation erwarten lassen

Der Übergang von Stufe A („es gibt mehr zu behandelnde Patienten als Behandlungsplätze“) zu Stufe B („es gibt gar keine Behandlungsplätze mehr“) kann wie z. B. im Rahmen der SARS-CoV-2-Pandemie unterschiedlich ausfallen. An einigen Standorten kam es innerhalb kürzester Zeit zu einem großen Patientenaufkommen im Sinne eines Massenanfalls. Eine Einschätzung nach Stufe A kann in einer solchen Situation von Nutzen sein. An vielen anderen Standorten ist allerdings viel mehr ein kontinuierlicher Patientenstrom zu verzeichnen. Hier ist zu erwarten, dass alle Behandlungsplätze belegt werden, ohne dass Behandlungskapazitäten durch erfolgreichen Abschluss der Behandlungen wieder zur Verfügung stehen. In diesem Szenario wäre der Algorithmus nach Stufe A nicht sinnvoll anzuwenden, sondern ein direkter Übergang in Stufe B angezeigt.

Die Festlegung der Priorisierungsstufe sowie des Scores sollte wünschenswerterweise durch eine übergeordnete Stelle (Ärztlicher Direktor, Kommunal‑, Landes- oder Bundesbehörde) festgelegt werden. Dafür gibt es aktuell aber weder ein geregeltes Verfahren, noch erscheint es realistisch, dass dieses in dynamischen Veränderungen während eines pandemischen Geschehens schnell genug handlungsfähig ist. Daher muss davon ausgegangen werden, dass das eingesetzte Triage-Team am ehesten den Überblick über die aktuelle Lage hat und selbstständig handelt.

Patienten, die nicht auf die Intensivstation aufgenommen bzw. deren Therapie auf der Intensivstation nicht weitergeführt werden kann, werden zur konservativen, supportiven oder palliativmedizinischen Weiterbetreuung auf die Normalstation verlegt.

## Anwendung und Dokumentationsbogen

**Figure 2:**
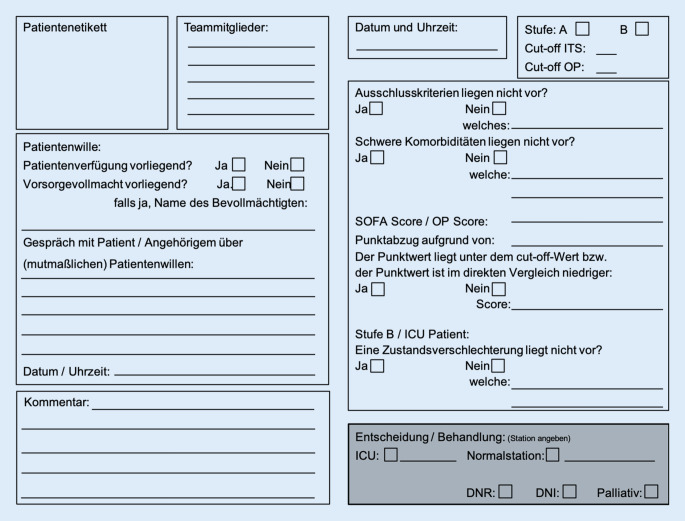

Zentraler Bestandteil des Konzeptes ist, dass ein behandlungsbedürftiger Patient auch in Pandemiezeiten von seinem Arzt nach allen Regeln des ärztlichen Handelns versorgt werden soll. Somit sind immer die medizinische Indikation, das realistisch erreichbare Therapieziel und der (mutmaßliche) Patientenwille für die entsprechende Situation entscheidend für den Beginn und die Fortführung medizinischer Maßnahmen. Sollte eine Priorisierung von übergeordneter Stelle als notwendig festgestellt worden sein, wechselt die individuelle Patientenperspektive zu einer bevölkerungsbezogenen Perspektive und es geht darum, so viel Menschenleben wie möglich zu erhalten. Die Priorisierung erfolgt durch das unabhängige und in die Therapie nicht eingebundene Triage-Team. Dieses dokumentiert zunächst den (mutmaßlichen) Patientenwillen (Abb. 2), um im Weiteren Ausschlusskriterien, Komorbiditäten und den Score zur Bestimmung der Prognose des Patienten zu erheben. In Stufe B ist auch bei allen Beatmungspatienten, die länger als 72 h intensivmedizinisch behandelt werden, der klinische Zustand bzw. die Prognose zur Priorisierung zu erfassen. Als Verschlechterung wird hier definiert, wenn der Patient nach einer initialen Stabilisierungsphase von 72 h a) im stationären Aufenthalt einen Herzstillstand erlitten hat, b) zusätzlich zum Aufnahmegrund zwei weitere Organversagen zeigt, c) eine signifikante Erhöhung des SOFA-Scores (>2 Punkte) berechnet wird oder d) keine Verbesserung der respiratorischen oder hämodynamischen Situation trotz aller Optimierungsbemühungen erreicht werden kann.

Das Triage-Team führt diese Dokumentation idealerweise einmal täglich bei allen beatmeten Patienten und je nach Patientenaufkommen regelmäßig in der Notaufnahme durch. Für das Team kommt es dann zu einer Priorisierungsliste, die Patienten vorrangig Beatmungsplätze zuweist. Patienten die nicht intensivmedizinisch behandelt werden können, werden auf eine Normalstation verlegt und je nach klinischem Zustand konservativ, supportiv oder palliativmedizinisch therapiert. Eine Verlegung von der Intensivstation wird erst dann durchgeführt, wenn ein Konflikt durch einen Patienten mit besserer Prognose entsteht, ein prophylaktisches Freihalten von Betten widerspricht den Grundsätzen dieses Konzeptes.

Eine Entscheidung gegen eine intensivmedizinische Behandlung schließt eine kurative Behandlung nicht grundsätzlich aus. Im Fall von Behandlungsabbrüchen und ansonsten fehlendem kurativem Ansatz erfolgt die palliativmedizinische Begleitung innerhalb der Fachklinik nach „best clinical practice“ und entsprechend palliativmedizinischen Standards.

## Möglichkeiten für Modifikationen

Das hier vorgestellte Konzept hat nach konstruktiver Diskussion ein Votum des Ethikkonsils der Uniklinik Köln erhalten. Bewusst sind an verschiedenen Stellen des Konzeptes Möglichkeiten zur Modifikation gegeben. Zum einen kann die Besetzung des Triage-Teams variiert werden bzw. durch die von ihrer Profession nicht definierten und frei zu nominierenden Personen des Ethikkonsils und des Ärztlichen Direktors Einfluss auf die Priorisierung nehmen. Vorgeschlagen ist eine Konsensbildung bzw. im Dissens Einbindung der größten klinischen Expertise.

Des Weiteren sind Ausschlusskriterien und Komorbiditäten nicht bis ins Detail definiert. So können diese durch Erfahrungen in der Anwendung, aber auch in der jeweiligen Situation durch das Triage-Team angepasst verwendet und unterschiedlich gewichtet werden. Es besteht auch die Möglichkeit, die Liste der Ausschlusskriterien bzw. Komorbiditäten zu bearbeiten und einzelne wegzulassen, zu ergänzen oder zu konkretisieren. Ebenfalls ist auch die Erweiterung der bewussten Bevorzugung oder Benachteiligung von Gruppen, so denn gewünscht, durch Punktbonus oder -malus möglich.

## Schlussbemerkungen

Das hier vorgestellte Priorisierungskonzept soll Medizinern ermöglichen, weiter ihrer Tätigkeit nachzukommen und ihre Patienten nach besten medizinischen Möglichkeiten zu behandeln. Erst im Anschluss an eine notwendige Notfallmaßnahme übernimmt ein unabhängiges Triage-Team, welches aufgrund der definierten Ressourcenknappheit die notwendige Priorisierung anhand eines vordefinierten Stufenmodells erstellt. Das Konzept basierend auf der Kombination der kurz- und langfristigen Prognose soll dabei ermöglichen, möglichst viele Menschenleben zu retten.
